# Enhancement of Mechanical and Thermal Properties of Polycaprolactone/Chitosan Blend by Calcium Carbonate Nanoparticles

**DOI:** 10.3390/ijms13044508

**Published:** 2012-04-10

**Authors:** Sanaz Abdolmohammadi, Samira Siyamak, Nor Azowa Ibrahim, Wan Md Zin Wan Yunus, Mohamad Zaki Ab Rahman, Susan Azizi, Asma Fatehi

**Affiliations:** 1 Department of Chemistry, Faculty of Science, University Putra Malaysia, 43400 UPM Serdang, Selangor, Malaysia; E-Mails: sam.siyamak@gmail.com (S.S.); mzaki53@gmail.com (M.Z.A.R.); susanazizi@yahoo.com (S.A.); a_fatehi_chem@yahoo.com (A.F.); 2 Department of Chemistry, Centre For Defence Foundation Studies, National Defence University of Malaysia, Sungai Besi Camp, 57000, Kuala Lumpur, Malaysia; E-Mail: wanmdzin@upnm.edu.my

**Keywords:** nanocomposites, calcium carbonate nanoparticles, polycaprolactone, chitosan, mechanical properties, thermal properties

## Abstract

This study investigates the effects of calcium carbonate (CaCO_3_) nanoparticles on the mechanical and thermal properties and surface morphology of polycaprolactone (PCL)/chitosan nanocomposites. The nanocomposites of PCL/chitosan/CaCO_3_ were prepared using a melt blending technique. Transmission electron microscopy (TEM) results indicate the average size of nanoparticles to be approximately 62 nm. Tensile measurement results show an increase in the tensile modulus with CaCO_3_ nanoparticle loading. Tensile strength and elongation at break show gradual improvement with the addition of up to 1 wt% of nano-sized CaCO_3_. Decreasing performance of these properties is observed for loading of more than 1 wt% of nano-sized CaCO_3_. The thermal stability was best enhanced at 1 wt% of CaCO_3_ nanoparticle loading. The fractured surface morphology of the PCL/chitosan blend becomes more stretched and homogeneous in PCL/chitosan/CaCO_3_ nanocomposite. TEM micrograph displays good dispersion of CaCO_3_ at lower nanoparticle loading within the matrix.

## 1. Introduction

Nanocomposites are an attractive class of materials providing novel performance. Due to some of their remarkable properties at low filler loading (less than 10 wt%), they are being increasingly adopted by industry whilst displacing the use of conventional filler materials. A variety of nanoscale fillers, including layered silicates and nanoparticles, are capable of enhancing mechanical and thermal properties of nanocomposites, including modulus, strength, impact performance, and heat resistance [1–3].

Among nanoparticles, CaCO_3_ is one of the most common and inexpensive inorganic fillers that has been used in the nanocomposite preparation process. Fine grade nano-sized precipitated CaCO_3_ (NPCC) has the potential to be an important functional filler in polymer systems such as polypropylene composites, poly (vinyl chloride) composites and poly (ethylene terephthalate) composites that are being produced [4–7]. It is a common practice to coat the surface of the NPCC with fatty acids such as stearic acid to reduce moisture absorption and particle agglomeration as well as to enhance dispersibility of the polar filler when incorporated into non-polar polymer melts [8].

Extensive studies on the reinforcing effect of nano- and micro-sized CaCO_3_ particles have been carried out in different polymer matrices, such as high density polyethylene (HDPE), nylon, polypropylene (PP), polylactide, acrylonitrile butadiene styrene (ABS), and thermoplastic polyurethane (TPU). Using nano-CaCO_3_ particles significantly improved the mechanical properties as well as the surface smoothness of nanocomposites [9–12].

Poly (ɛ-caprolactone) (PCL) is a biodegradable aliphatic polyester regularly prepared through ring opening of ɛ-caprolactone in the presence of metal alkoxides (aluminum isopropoxide, tin octoate, *etc.*) [13]. Some particular features of PCL include flexibility, low Tg (−61 °C), and low melting point (65 °C), comparatively high price, and a fairly long biodegradability cycle [14,15].

Based on its biodegradable characteristics, PCL is broadly applied in environmental (e.g., soft compostable packaging) and biomedical (e.g., blood bags and catheters) applications [16].

To overcome some of the PCL properties’ drawbacks, such as high price and long biodegradability cycles, in some cases PCL is blended with a cheaper biodegradable natural polymer, such as starch, cellulous, or chitin and chitosan [15–21].

Chitosan is a polysaccharide, composed mainly of b-(1,4)-linked 2-deoxy-2-amino-D-glucopyranose units, which is the deacetylated product of chitin (poly (*N*-acetyl-D-glucopyranose)), being the second most plentiful natural based biopolymer after cellulose [22–25]. A waste product, chitosan is an antibacterial, non-toxic, biocompatible, and edible biodegradable polymer with good mechanical properties, biocompatibility and biodegradability, and has been widely used for several decades in different fields of studies, such as water engineering as part of a filtration process (removing phosphorus, heavy minerals, and oils from the water), food packaging film (edible coatings or films used for extending the shelf life of foodstuffs of, for example, fruit, meat, and fish and seafood), artificial skin and bone substitutes [26,27].

Chitosan, due to its antibacterial and biodegradable properties, mixed with PCL, a flexible material, can be broadly used for antibacterial packaging purposes.

However, PCL and chitosan are immiscible, because PCL is a hydrophobic polyester, whereas chitosan is a hydrophilic polysaccharide. Consequently, their blends suffer from some weaknesses in thermal and mechanical properties, making them uncompetitive for many applications [28].

Many studies have focused on improving the compatibility between these two phases, either by adding a third component or by stimulating a chemical reaction leading to a modification of the polymer interface. Wu has investigated the effect of grafting acrylic acid onto PCL on the properties of PCL/chitosan blend [16]. Liu *et al.* used chemical reaction to prepare the biosynthetic graft copolymers of chitosan with PCL, a product applicable in a variety of purposes [29].

So far, no study has been undertaken to investigate the use of CaCO_3_ nanoparticles in polycaprolactone/chitosan composites. The principal goal of this research is to improve the mechanical and thermal properties of PCL/chitosan composites by adding CaCO_3_ nanoparticles as a nano-filler.

## 2. Results and Discussion

### 2.1. Results of Nanoparticle Characterization

#### 2.1.1. Electron Microscopic Analysis

**Figure 1:**
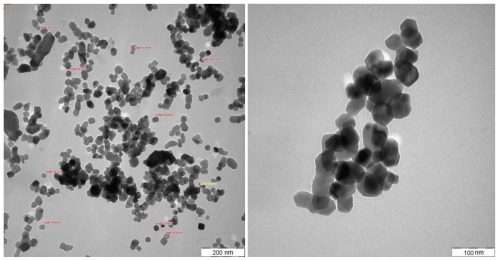
TEM micrographs of CaCO_3_ nanoparticles.

A TEM micrograph of CaCO_3_ nanoparticles is shown in Figure 1. As illustrated in Figure 1, nanoparticles have cubic shape with an average size of 62 nm calculated from the size of 10 randomly chosen particles.

#### 2.1.2. Fourier Transform Infrared Characterization (FTIR)

**Figure 2:**
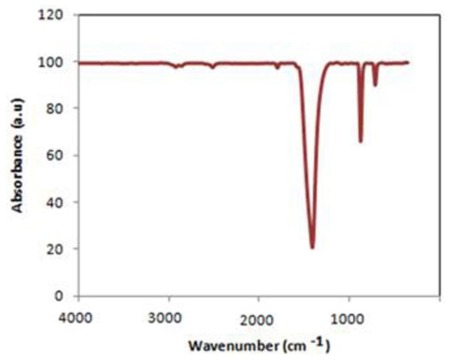
FTIR spectrum of the nano CaCO_3_.

From the FTIR spectrum of CaCO_3_ nanoparticles illustrated in Figure 2, it can be seen that the nano CaCO_3_ had adsorption bands at 2950–2840 cm^−1^, corresponding to the vibration mode of C–H of stearic acid, and also 707, 873 and 1418 cm^−1^ corresponding to the in-, out-plane bending and asymmetrical stretching vibration peaks of O-C-O, respectively. They all are characteristic peaks in calcite [30,31].

#### 2.1.3. Thermogravimetric Analysis

**Figure 3:**
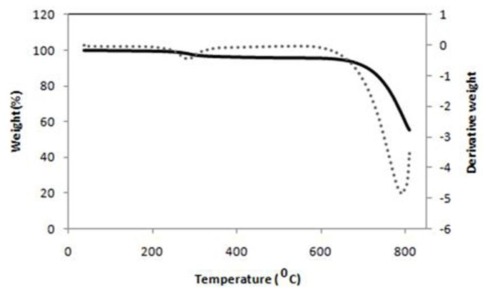
TGA and DTG (Thermal degradation properties) curve of CaCO_3_ nanoparticles.

The weight loss and derivative weight of nanoparticles as a function of temperature are shown in Figure 3; two stages of degradation of nano-sized CaCO_3_ are displayed. The first stage accruing at 260 °C belongs to degradation of stearic acid, and the second stage at 720 °C corresponds to the decomposition of CaCO_3_ itself [31]. This result specifies that processing temperature for melt blending PCL/chitosan/nano CaCO_3_ should be lower than 260 °C.

### 2.2. Results of Nanocomposites Characterization

#### 2.2.1. Mechanical Properties of Nanocomposites

As illustrated in Table 1, by increasing the amount of CaCO_3_ nanoparticles, tensile modulus increases. The increase in tensile modulus must be caused by rigidity of the filler and strong interaction between the polymer and filler due to the large interfacial area between particles [32,33]. The enhanced composite modulus as a result of nanofiller loading has also been reported by several research groups [34,35].

Increasing CaCO_3_ nanoparticle content enhances the tensile strength and peaks at 1 wt% of nano-sized CaCO_3_ loading, due to good dispersion of nanoparticles into the matrix, yielding a higher reinforcement effect. Acting similarly to a compatibilizing agent, nanoparticles enhance the dispersion of materials and the interfacial adhesion within the matrix. By further loading of nano-sized CaCO_3_, the tensile strength gradually decreases as a result of the agglomeration of filler particles at higher amounts [36]. Particle agglomeration flaws the material’s surface, which restrains stress movement, and eventually results in decreased tensile strength [35].

By loading different amounts of nano-sized CaCO_3_, the elongation at break decreases except for 0.5 and 1 wt% of nano CaCO_3_. The higher elongation at break at lower nano-sized CaCO_3_ loading is due to the good dispersion of nanoparticles within the matrix [37]. However, at higher filler loading, a large amount of agglomeration was more apparent, due to the high surface energy of nanoparticles, thus contributing to the lower elongation at break [38,39].

The tensile results imply a good dispersion of the CaCO_3_ nanoparticles at 1 wt% in the matrix; the finding is also approved by SEM image.

#### 2.2.2. Thermal Properties of Nanocomposites

**Figure 4:**
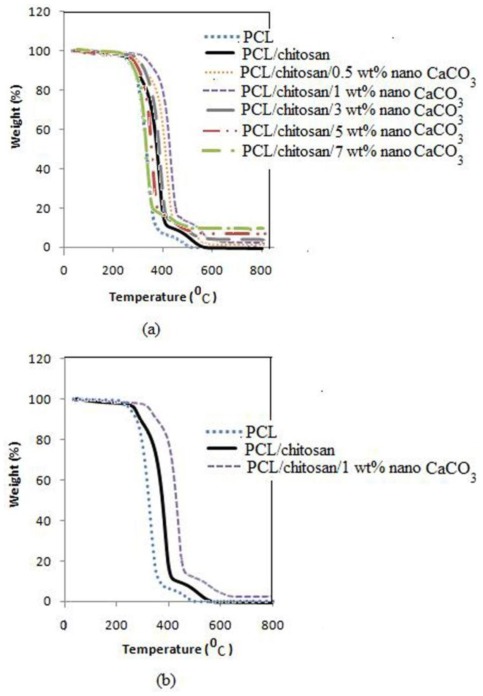
TGA curves of: (**a**) PCL, and PCL/chitosan with different amount of CaCO_3_ nanoparticles; (**b**) pristine composite (PCL/chitosan) and nanocomposite with highest thermal stability.

**Figure 5:**
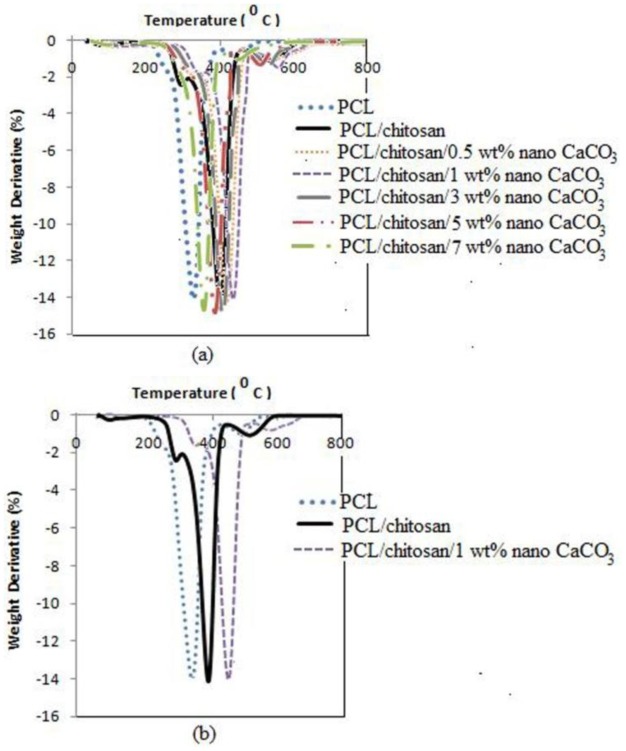
DTG curves of: (**a**) PCL, and PCL/chitosan with different amounts of CaCO_3_ nanoparticles; (**b**) pristine composite (PCL/chitosan) and nanocomposite with highest thermal stability.

TGA and DTG thermograms of PCL, PCL/chitosan blend and its nanocomposites are displayed in Figures 4 and 5, respectively. Figure 4 illustrates samples weight losses as the temperature increases. CaCO_3_ nanoparticles raise the temperature at which the degradation process initiates. As shown in Figure 4b, the onset decomposition temperature of 223 °C for PCL and 231.45 °C for PCL/chitosan composite increases to 258.76 °C by loading 1 wt% nano-sized CaCO_3_. Figure 5b exhibits two stages of degradation of the PCL/chitosan blend. The first stage at T_max 1_ = 283 °C corresponds to chitosan backbone degradation resulting from saccharide ring dehydration, and the second stage at T_max 2_ = 382 °C corresponds to PCL decomposition [28].

Figure 5b shows that the second stage occurring at T_max 2_ increases considerably from 382.89 °C for the PCL/chitosan composite to 446.87 °C after the addition of 1 wt% CaCO_3_ nanoparticles. The nanocomposites possess higher thermal stability compared to the PCL/chitosan composite, due to better adhesion between reinforced CaCO_3_ nanoparticles and matrix and the hindered diffusion of volatile decomposition products resulting from homogeneous dispersion of nanoparticles within the nanocomposites [31,32].

#### 2.2.3. Morphology Observation

Fractured surface morphology of the PCL/chitosan blend, as well as its nanocomposite, was studied by SEM.

**Figure 6:**
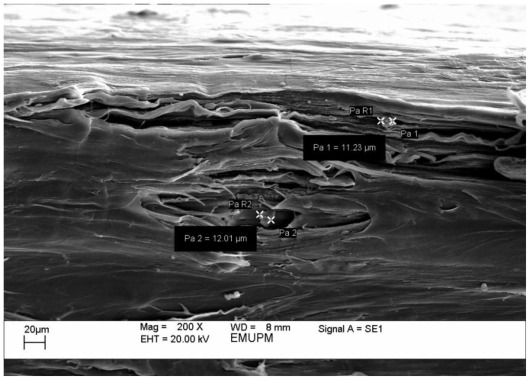
SEM micrograph of PCL/chitosan composite.

Figure 6 shows that the PCL/chitosan composite possesses a stretched and porous surface. The low interaction between the two components and the lack of interfacial adhesion are the underlying reasons. The unmixed chitosan particles sizing (11.23, 12.01 μm) are illustrated in the Figure 6.

**Figure 7:**
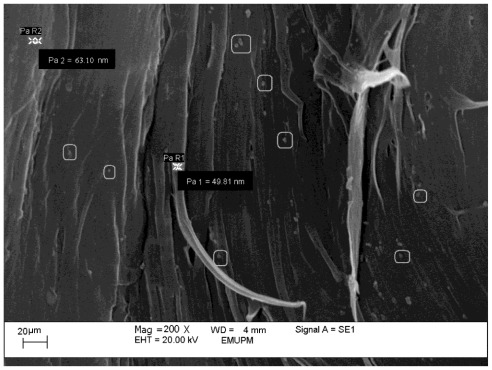
SEM micrograph of PCL/chitosan/1 wt% CaCO_3_ nanoparticles.

A large number of small discrete nanoparticles on the fractured surface of the PCL/chitosan/CaCO_3_ nanocomposite are exhibited in the Figure 7. The sizes of the randomly selected CaCO_3_ nanoparticle in PCL/chitosan/CaCO_3_, as displayed in Figure 7, are 49.81 and 63.10 nm. This observation compared to the average of 10 solely CaCO_3_ nanoparticle measured size of 62 nm and shown in Figure 1, confirming that the particles highlighted in Figure 7 are CaCO_3_ nanoparticles. As shown in the Figure 7 by adding 1 wt% nano-sized CaCO_3_ the surface becomes smoother, the dispersion of the nanoparticle is improved and the interfacial interaction between nanoparticles and matrix becomes stronger [39].

**Figure 8:**
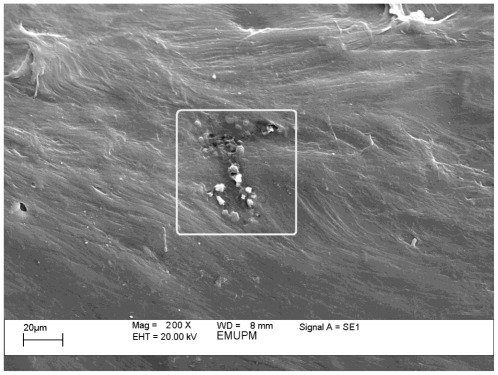
SEM micrograph of PCL/chitosan/5 wt% CaCO_3_ nanoparticles.

As Figure 8 shows, at a higher rate of filler loading (about 5 wt%), a higher rate of agglomeration occurs, lowering the tensile strength. These results are in line with measured mechanical results.

**Figure 9:**
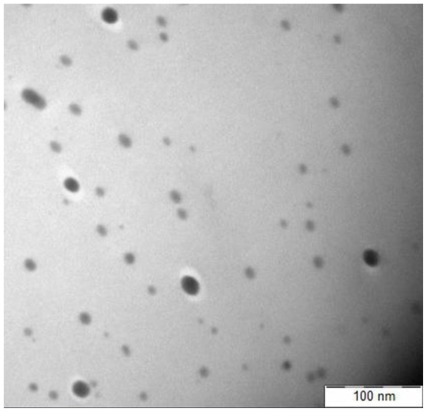
TEM micrograph of PCL/chitosan/1 wt% CaCO_3_ nanoparticles.

**Figure 10:**
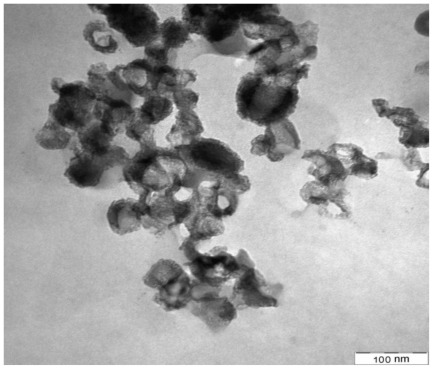
TEM micrograph of PCL/chitosan/5 wt% CaCO_3_ nanoparticles.

TEM was applied to investigate the dispersion of nanoparticles within the matrix. Figure 9 shows good dispersion of 1 wt% of nano CaCO_3_; however, Figure 10 displays the higher rate of agglomeration which is predominantly formed at nanometer scale by increasing the nanoparticle’s content to 5 wt%. This is due to the fact that the distance between nanoparticles shrinks at higher filler loadings, making it more likely for nanoparticles to reunite and, as a consequence, reduce the dispersion of the CaCO_3_ particles, resulting in more aggregation.

## 3. Experimental Section

### 3.1. Materials

Commercial grade poly (ɛ-caprolactone) (CAPA6500) was supplied from Solvay, Warrington, UK. Chitosan with a degree of deacetylation of 85% and average molecular weight of 345,500 g·mol^−1^ was obtained by the Malaysian Nuclear Agency and dried at 50 °C for 3 days. Calcium carbonate nanoparticles with surface coating of stearic acid were purchased from Malaysian Nuclear Agency.

### 3.2. Preparation of Nanocomposites

PCL/chitosan nanocomposites were carried out by a melt blending method using a Brabender Plastograph D28033 Berman with a rotor speed of 50 rpm, reaction time of 20 min and mixing temperature of 100 °C. PCL pellets were first added to the mixing chamber, which had been heated and stabilized at 100 °C. Then, after 2 min of heating, chitosan was added in four equal portions at 2-min intervals to the molten PCL. This was followed by the additions of 0.5, 1, 3, 5, or 7 wt% of CaCO_3_ nanoparticles into the PCL/chitosan (90/10 wt%). The CaCO_3_ nanoparticles were added slowly over a period of 10 min into the chamber to give well dispersed ternary composites.

To prepare a sheet of composite with a thickness of 1mm, compression molding was carried out by hot pressing of 1.08 × 10^7^ Pa at 100 °C for 5 min, and then cold pressing at room temperature for 5 min under the same pressure.

As shown in Table 2, by increasing the amount of chitosan beyond 10 wt%, tensile strength and elongation at break decrease, while the tensile modulus increases, making the composite stiff and rigid. Therefore, PCL/chitosan (90/10 wt%) was chosen as the optimum ratio with the tensile strength, flexibility and elongation at break most in line with the composite’s potential application in packaging.

### 3.3. Characterizations

The infrared spectra of samples were recorded on a Perkin-Elmer FTIR (model spectrum 100 series). FTIR spectra tests were run at ambient temperature using a KBr disk method at wave number range of 400 to 4000 cm^−1^, resolution of 4 cm^−1^.

TEM micrographs were obtained by a Hitachi H-7100 at an accelerating voltage of 200 kV. The nano CaCO_3_-acetone suspension was prepared and diluted to the correct concentration in an ultrasonic bath for 10 min and then air dried by dropping onto the TEM sample copper grid, and ultrathin sections of nanocomposite sample were prepared at −120 °C using a microtome equipped with a diamond knife, and then placed on a copper grid.

Tensile tests were performed with an Instron Universal Testing Machine (4302 series IX, type V) according to the guidelines of ASTM Standard Method D638-5. The dumbbell-shaped specimens were prepared by a hydraulic cutter. A load cell of 1.0 kN was applied at a constant crosshead speed of 5 mm/min at room temperature. The average of five measurements of tensile determination was calculated.

Thermogravimetric analysis was carried out using a Perkin Elmer Thermal Analyzer model TGA7, on 8–10 mg weight samples in temperature range of 35–800 °C with heating rate of 10 °C/min, under nitrogen atmosphere with a flow rate of 20 mL/min.

Scanning electron microscopy (SEM) was carried out using Leo 1455 VP SEM. The samples were coated with gold by Bal-Tec SDC005 sputter coater.

## 4. Conclusions

PCL/chitosan nanocomposites with various amounts of CaCO_3_ nanoparticles were successfully prepared by a melt intercalation method. The tensile modulus increases by adding CaCO_3_ nanoparticles into the composite. The increase in the modulus must be caused by the rigidity of the filler and strong interactions between the polymer and filler due to the large interfacial area between particles. The tensile strength and elongation at break increase by loading up to 1 wt% of nano-sized CaCO_3_, and then decrease with the increase of nanoparticle content. This incident occurred due to optimal dispersion of nanoparticles within the matrix and intercalation of nanoparticles by PCL/chitosan at low filler content. However, at high filler loading, due to the agglomeration of nanoparticles into the matrix, the elongation at break decreases.

TGA characterization shows significantly improved thermal stability of PCL/chitosan/nano-CaCO_3_ compared to that of PCL/chitosan composite. Compared to that of the pristine composite, thermal degradation temperature increases up to 64 °C at 1 wt% of nano-sized CaCO_3_ content in PCL/chitosan nanocomposite. The nanocomposites possess higher thermal stability compared to the PCL/chitosan composite due to better adhesion between reinforced CaCO_3_ nanoparticles and matrix and the hindered diffusion of volatile decomposition products resulting from homogeneous dispersion of nanoparticles within the nanocomposites. SEM micrographs show that the nanocomposite becomes more homogeneous than the former composite. However, at higher filler loadings, a higher rate of nanoparticle agglomeration occurs, lowering the tensile strength. Good dispersion of CaCO_3_ nanoparticles into the matrix results in increased interfacial adhesion and a smoother surface in PCL/chitosan/nano-CaCO_3_ nanocomposite.

TEM micrographs show good dispersion of nanoparticles within the matrix at 1 wt% nano CaCO_3_, whereas at higher nanoparticle CaCO_3_ loading (5 wt%), due to the decrease in distance between nanoparticles, the dispersion of the CaCO_3_ reduced, hence increasing agglomeration.

## Figures and Tables

**Table 1:** Tensile properties of PCL/chitosan composite with different amounts of nano CaCO_3_.

Composite	Tensile Strength (MPa)	Elongation at Break (%)	Modulus (MPa)
PCL/chitosan	15.05 ± 0.84	848.00 ± 6.87	160.26 ± 4.15
PCL/chitosan/0.5 wt% nano CaCO_3_	17.31 ± 0.92	915.00 ± 7.84	190.58 ± 3.20
PCL/chitosan/1 wt% nano CaCO_3_	20.18 ± 0.96	1215.00 ± 8.35	214.91 ± 3.53
PCL/chitosan/3 wt% nano CaCO_3_	14.65 ± 0.73	751.00 ± 6.54	231.80 ± 3.89
PCL/chitosan/5 wt% nano CaCO_3_	12.16 ± 0.67	460.00 ± 5.31	247.21 ± 5.36
PCL/chitosan/7 wt% nano CaCO_3_	9.18 ± 0.54	124.00 ± 4.57	278.60 ± 4.53

**Table 2:** Tensile properties of PCL/chitosan composite with different proportion.

PCL/Chitosan	100/0	90/10	80/20	70/30	60/40	50/50
**Tensile Strength**	20.59	15.05	13.23	10.80	8.50	4.89
**Elongation at break (%)**	1300.00	848.60	505.47	320.10	208.12	87.34
**Tensile Modulus**	130.26	160.26	168.50	178.32	186.64	195.10
